# CAD204520 Targets *NOTCH1* PEST Domain Mutations in Lymphoproliferative Disorders

**DOI:** 10.3390/ijms25020766

**Published:** 2024-01-07

**Authors:** Luca Pagliaro, Elisa Cerretani, Federica Vento, Anna Montanaro, Lucas Moron Dalla Tor, Elisa Simoncini, Mariateresa Giaimo, Andrea Gherli, Raffaella Zamponi, Isotta Tartaglione, Bruno Lorusso, Matteo Scita, Filomena Russo, Gabriella Sammarelli, Giannalisa Todaro, Enrico Maria Silini, Gian Matteo Rigolin, Federico Quaini, Antonio Cuneo, Giovanni Roti

**Affiliations:** 1 Department of Medicine and Surgery, University of Parma, 43126 Parma, Italy; luca.pagliaro@unipr.it (L.P.); anna.montanaro@unipr.it (A.M.); lucas.morondallator@unipr.it (L.M.D.T.); elisa.simoncini@unipr.it (E.S.); mariateresa.giaimo@unipr.it (M.G.); andrea.gherli@unipr.it (A.G.); zamponir@gmail.com (R.Z.); bruno.lorusso@unipr.it (B.L.); enricomaria.silini@unipr.it (E.M.S.); federico.quaini@unipr.it (F.Q.); 2 Translational Hematology and Chemogenomics (THEC), University of Parma, 43126 Parma, Italy; crrlse@unife.it (E.C.); federica.vento@unife.it (F.V.); isotta.tartaglione@studenti.unipr.it (I.T.); 3 Hematology and BMT Unit, University Hospital of Parma, 43126 Parma, Italy; frusso@ao.pr.it (F.R.); gsammarelli@ao.pr.it (G.S.); giannat@gmail.com (G.T.); 4 Department of Medical Science, University of Ferrara, 44121 Ferrara, Italy; matteo.scita@edu.unife.it (M.S.); gianmatteo.rigolin@unife.it (G.M.R.); cut@unife.it (A.C.); 5 Hematology Unit, University Hospital of Ferrara, 44121 Ferrara, Italy

**Keywords:** NOTCH1, target therapy, *NOTCH1* PEST domain mutations, lymphoproliferative disorders, chronic lymphocytic leukemia, mantle cell lymphoma, synergy treatment

## Abstract

*NOTCH1* PEST domain mutations are often seen in hematopoietic malignancies, including T-cell acute lymphoblastic leukemia (T-ALL), chronic lymphocytic leukemia (CLL), splenic marginal zone lymphoma (SMZL), mantle cell lymphoma (MCL), and diffuse large B-cell lymphoma (DLBCL). These mutations play a key role in the development and progression of lymphoproliferative tumors by increasing the Notch signaling and, consequently, promoting cell proliferation, survival, migration, and suppressing apoptosis. There is currently no specific treatment available for cancers caused by *NOTCH1* PEST domain mutations. However, several NOTCH1 inhibitors are in development. Among these, inhibition of the Sarco-endoplasmic Ca^2+^-ATPase (SERCA) showed a greater effect in *NOTCH1*-mutated tumors compared to the wild-type ones. One example is CAD204520, a benzimidazole derivative active in T-ALL cells harboring *NOTCH1* mutations. In this study, we preclinically assessed the effect of CAD204520 in CLL and MCL models and showed that *NOTCH1* PEST domain mutations sensitize cells to the anti-leukemic activity mediated by CAD204520. Additionally, we tested the potential of CAD204520 in combination with the current first-line treatment of CLL, venetoclax, and ibrutinib. CAD204520 enhanced the synergistic effect of this treatment regimen only in samples harboring the *NOTCH1* PEST domain mutations, thus supporting a role for Notch inhibition in these tumors. In summary, our work provides strong support for the development of CAD204520 as a novel therapeutic approach also in chronic lymphoproliferative disorders carrying *NOTCH1* PEST domain mutations, emerging as a promising molecule for combination treatment in this aggressive subset of patients.

## 1. Introduction

Notch1 is a highly conserved signaling pathway that mediates several processes, from the physiological mechanism of embryogenesis and tissue commitment to hereditary syndromes and oncological diseases [1]. NOTCH1 protein subdomains are the target of recurrent mutations leading to the aberrant activation of oncogenic downstream signals. For instance, mutations in the heterodomain (HD) and juxta-membrane expansion domain (JME) are the main hotspot sites for class 1 and class 2 nucleotide variations causing an enhanced instability of NOTCH1 and constitutive activation of the Notch pathway targets [2,3]. These mutations recur in several cancers, such as T-cell acute lymphoblastic leukemia (T-ALL), breast cancer, and other solid tumors [4]. In addition, a second hotspot region involves the *NOTCH1* exon 34 in the PEST (rich in proline (P), glutamic acid (E), serine (S), and threonine (T)) sequence. The PEST domain is a substrate of the E3-ubiquitin ligase for ubiquitination, which is needed to maintain the stability and the turnover of the intracellular NOTCH1 domain (N1-ICD) [4]. Short insertions or deletions in this hotspot disrupt the binding site of the E3-ubiquitin ligase, reducing the cleavage and the inactivation of N1-ICD [2]. Isolated PEST mutations such as the 2 bp-deletion p.(Pro2514Argfs*4), count for 15–20% mutations of T-ALL, 5 to 15% of mature B-cell malignancies, such as chronic lymphocytic leukemia (CLL) and mantle cell lymphoma (MCL), splenic marginal zone lymphoma (SMZL) and less than 5% of diffuse large B-cell lymphoma (DLBCL) [2,5,6,7,8,9].

The role of *NOTCH1* in oncogenesis sets the conditions for the development of Notch inhibitors. One example includes modulators of the γ-secretase complex (GSI), which is required for releasing N1-ICD from transmembrane NOTCH1 receptor (N1-TM). Unfortunately, so far, these approaches have shown no selectivity on mutant vs. wild-type (WT) proteins, limiting their clinical transition for *NOTCH1*-mutated malignancies [10].

Previous work from our group and Dr. Stegmaier’s laboratory identified the Sarco-endoplasmic Ca^2+^-ATPase (SERCA) as a potential therapeutic target in *NOTCH1*-mutated cancers, overcoming the innate limitations associated with GSI [11] that equally target WT and mutated NOTCH1 proteins [11,12]. Thapsigargin and other SERCA inhibitors impair the trafficking of full-length NOTCH1 (N1-FL) toward the cellular membrane limiting the access of preprocessed NOTCH polypeptides to the γ-secretase complex. This effect is more impactful in Notch-mutated cancers [12] or leukemia subtypes highly dependent on this signal [13].

Continuous development efforts in the field have led to the identification of a SERCA binding site that is permissive to pharmacological inhibition (CAD204520) [14]. Unlike the binding site occupied by thapsigargin, the lock-in of this residue does not cause significant changes in Ca^2+^ homeostasis, evolving into a model for a medicinal chemistry effort to optimize the design of SERCA inhibitors with improved tolerability and efficacy [14].

While the role of *NOTCH1* mutations in prognosis has not been established for several tumors and, for others, it remains unclear [6,7,15,16], PEST mutations identify a subset of lymphoproliferative disorders characterized by a poor prognosis, chemorefractoriness, and higher incidence of Richter’s transformation in the case of CLL [6,7,15,16,17]. Hence, in this work, we extend our experience in the field by providing the context for targeting the Notch1 pathway in tumors with isolated PEST domain mutations.

## 2. Results

### 2.1. Patient Cohort Characterization

**Figure 1:**
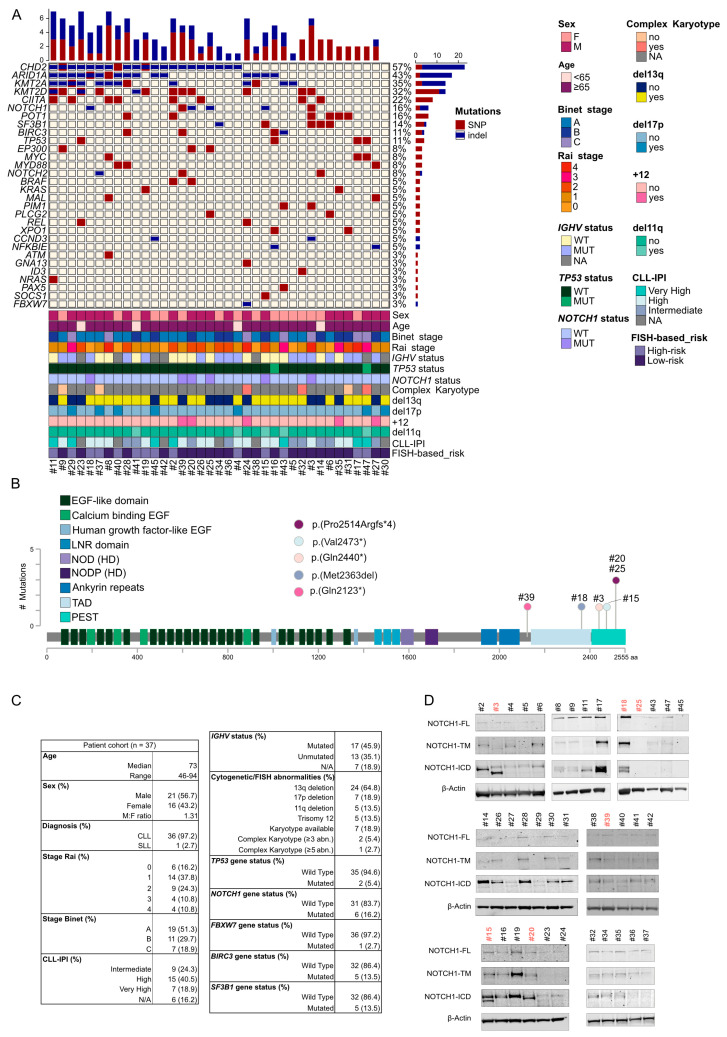
*NOTCH1* mutational status in chronic lymphocytic leukemia (CLL) primary samples and characteristics of the patient cohort: (**A**) The OncoPrint illustrates the distribution of gene mutations affecting individual samples. Single nucleotide polymorphisms (SNPs) are represented in red, and insertions/deletions (indels) are in blue. Each row in the OncoPrint displays the percentage distribution of relative gene mutations in the entire cohort (shown in the right histogram panel). Each column represents the total number of mutations for each patient, with a specific indication of the mutation type (upper histogram panel; red: SNP, blue: indel). The OncoPrint also provides relevant clinical, genetic, molecular, and prognostic characteristics of the patient samples collected for this study (lower panel). (**B**) The structure of the human NOTCH1 protein is depicted, with each colored block representing an exon. The PEST domain illustrates the distribution of PEST mutations found in CLL patient samples. (**C**) Patient characteristics for the collected CLL primary samples. (**D**) Western immunoblotting results display the expression of unprocessed full-length NOTCH1 precursor (FL), furin-processed NOTCH1 transmembrane subunit (TM), and cleaved intracellular domain (ICD) in CLL primary samples. β-Actin serves as the loading control. *NOTCH1*-mutated patient samples are indicated in light red.

Isolated PEST mutations most frequently occur in CLL and MCL, as such they represent an ideal model to investigate the development of Notch1-targeted therapies. From October 2021 to August 2022, we collected 37 leukemic cell samples from patients affected by CLL. The cohort included 21 males and 16 females (male-to-female ratio: 1.31) with a median age of 73 years (range: 46–94). All cases were classified according to the Rai–Binet staging classification, the CLL-international prognostic index (CLL-IPI), and characterized by genetic and molecular genomics approaches including cytogenetics, fluorescence in situ hybridization (FISH), immunoglobulin heavy chain variable (*IGHV*) gene mutation status, and mutational fingerprint by next-generation sequencing (NGS) (Figure 1A and Table S1). The median lymphocyte count was 54,460/μL (range: 10,070–286,000/μL). Twenty-nine patients out of thirty-seven (78.3%) were treatment-free, seven (18.9%) received one line of therapy, and three were undergoing ibrutinib treatment at the time of sample collection (Table S1). We sequenced 54 genes involved in lymphomagenesis, and this analysis revealed a frequency of *NOTCH1* mutations in 16% of cases, 6 out of 37, in line with previous findings [5]. We observed five different *NOTCH1* mutations in the exon 34, encoding for the PEST domain (Figure 1B). Two patients carried a p.(Pro2514Argfs*4) mutation, one exhibited a p.(Val2473), one a p.(Gln2440del), and one a mutation occurring in the ankyrin repeats motif p.(Gln2123*). These mutations are common in this disease [18]. We also recorded a rarer three nucleotide deletion, p.(Met2363del), occurring in the transactivation domain (TAD) and causing a frameshift deletion. A CLL patient showed an *FBXW7* mutation p.(Met404Ilefs*3) expected to increase the N1-ICD half-life [19]. The most recurrent mutations were observed in the *CHD2*, *ARID1A*, *KMT2A*, and *KMT2D* genes with frequencies of 56.7%, 43.2%, 35.1%, and 32.4%, respectively. Additionally, we identified the presence of other unfavorable mutations in the *BIRC3* and *SF3B1* genes, both at a frequency of 13.5%. Cytogenetic testing was examined on seven patients (18.9%), three of whom exhibited a complex karyotype. According to the FISH analysis, deletions of 13q, 17p, 11q, and trisomy of chromosome 12 were found in 64.8%, 18.9%, 13.5%, and 13.5% of cases, respectively (Figure 1C and Table S2). *TP53* mutations affected two out of thirty-seven patients (5.4%) in our series, consistent with previous reports [20,21,22]. Additionally, two other patients exhibited a minor *TP53*-mutated subclone with a variant allele fraction (VAF) of 4.5 and 7.7%, respectively. Furthermore, in one of these cases, there was a concurrent presence of del(17p).

Previous studies have suggested that the NOTCH1 activation, monitored by a monoclonal antibody that targets N1-ICD may occur also in the absence of a gain-of-function mutation [23]. In our cohort, this appears to be true in 67% of the cases, confirming the presence of samples with no detectable mutation by NGS but with N1-ICD expression and suggesting differences in the turnover of the intracellular domain (Figure 1D).

### 2.2. CAD204520 Suppresses Leukemia Growth and Notch1 Signaling in PEST-Mutated Cells

**Figure 2:**
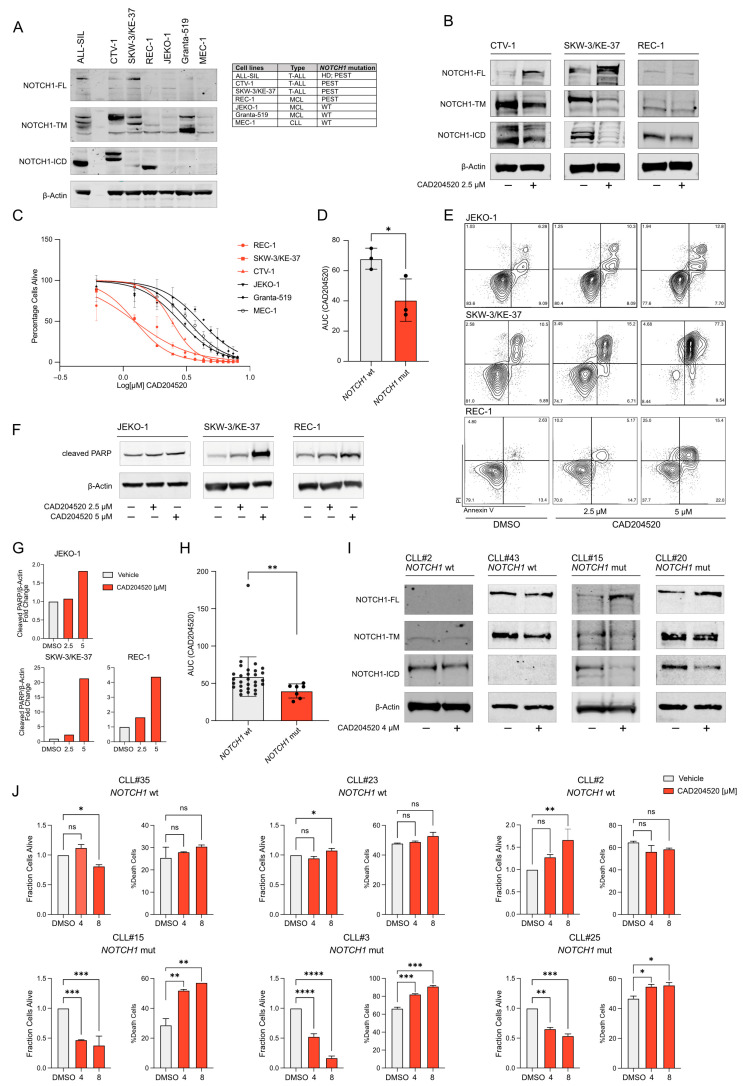
CAD204520 inhibits Notch1 signaling and impairs cell growth in PEST-mutated lymphoproliferative malignancies: (**A**) Protein expression of *NOTCH1* processed isoforms in a panel of T-ALL, MCL, and CLL cell lines. β-Actin was used as a loading control. (FL: full-length unprocessed precursor; TM: transmembrane; ICD: intracellular domain). The table shows *NOTCH1* mutational status in the cell lines. (**B**) Effect of CAD204520 treatment for 24 h on Notch1 trafficking and activation in cell lines (CTV-1, SKW-3/KE-37, REC-1) with PEST domain mutations. β-Actin was used as a loading control. (**C**) Effect of CAD204520 treatment on cell viability after 72 h in *NOTCH1* PEST-mutated (REC-1, SKW-3/KE-37, CTV-1) and *NOTCH1* WT (JEKO-1, Granta-519, MEC1) cell lines. Error bars denote ± SD of a minimum of two replicates. (**D**) Comparison of the area under the curve (AUC) values after CAD204520 treatment of *NOTCH1*-mutated and WT cell lines. Statistical significance was determined by a non-parametric *t*-test (* *p* < 0.05). (**E**) Effect of CAD204520 treatment on the induction of apoptosis. Annexin V/propidium iodide staining of MCL cells after 48 h of treatment with the indicated concentrations of CAD204520. A minimum of 20000 events was collected for each condition. (**F**) Western immunoblot showing the expression of cleaved PARP in *NOTCH1* WT (JEKO-1) and mutated (SKW-3/KE-37 and REC1) cell lines treated at the indicated concentrations of CAD204520 for 24 h. β-Actin was used as a loading control. (**G**) Densitometric quantification of indicated proteins in JEKO-1, SKW-3/KE-37, and REC-1 cells treated with indicated doses of CAD204520, as in Figure 2F. (**H**) Combined scatter and bar plot representing the AUC values of CAD204520 treatment in CLL primary samples with or without Notch1 activating pathway mutations. Statistical significance was determined using a non-parametric *t*-test (** *p* < 0.01). (**I**) Effect of CAD204520 treatment after 24 h on Notch1 trafficking in CLL primary samples. β-Actin was used as a loading control. (**J**) Histogram plots showing the percentage of live and dead cells in 6 different samples (top: 3 *NOTCH1* WT samples; bottom: 3 *NOTCH1*-mutated samples) after 72 h of treatment with CAD204520 at indicated concentrations. Results were obtained with a luminescence-based and a flow cytometric assay, respectively. Error bars denote the SD of a minimum of two replicates. Statistical significance among groups was determined by a one-way ANOVA using Dunnett’s correction for multiple comparison testing (* *p* < 0.05; ** *p* < 0.01; *** *p* < 0.001; **** *p* < 0.0001).

To test the antiproliferative effect of CAD20520 in PEST-mutated lymphoproliferative disorders, we used the following cell line models: T-ALL (ALL-SIL, CTV-1, SKW-3/KE-37), MCL (REC-1, JEKO-1, Granta-519) and CLL (MEC-1). Cell lines carrying PEST domain mutations, such as CTV-1, SKW-3/KE-37, and REC-1, showed N1-ICD expression compared to the WT ones (Figure 2A).

SERCA inhibitors block Notch trafficking and impede Notch proteins’ delocalization on the cellular surface [11,14]. This effect can be monitored by several approaches including immunofluorescence analysis and western blotting [14]. SERCA inhibitors are expected to decrease the level of the *NOTCH1* transmembrane subunit (N1-TM) (~110 kDa) while accumulating the unprocessed *NOTCH1* (N1-FL) (~270 kDa) polypeptides in the endoplasmic reticulum. Consistently, PEST-mutated tumors responded to this hypothesis, as shown in Figure 2B. If Notch signaling sustains the growth of leukemia cells, its inhibition ultimately leads to a decrease in cellular proliferation and cell death. We compared PEST-mutated tumor models (CTV-1, SKW-3/KE-37, and REC-1) to WT ones (JEKO-1, Granta-519, MEC-1) and showed that cell lines carrying the PEST mutation were more sensitive to CAD204520 inhibition (Figure 2C,D) with increasing apoptosis after treatment (Figure 2E–G).

We next extended these observations to clinical samples carrying *NOTCH1* mutations. In these samples, we confirmed an increased level of sensitivity in cells with mutations compared to WT (Figure 2H). N1-FL accumulates, N1-TM, and N1-ICD decreased upon CAD204520 treatment in mutated cells, according to the mechanism described above (Figure 2I). We also observed a difference in viability and anti-proliferative response to CAD204520 between WT and mutated samples through an ATP-based viability assay and flow cytometry analysis (Figure 2J).

Overall, our data suggest that CAD204520 inhibits *NOTCH1* PEST mutations in both cell lines and in primary CLL samples, retaining the advantageous anti-tumor effect on mutated WT cells.

### 2.3. CAD204520 Treatment Exerts Preferential Anti-Notch1 Efficacy in a Xenograft Lymphoma Model

**Figure 3:**
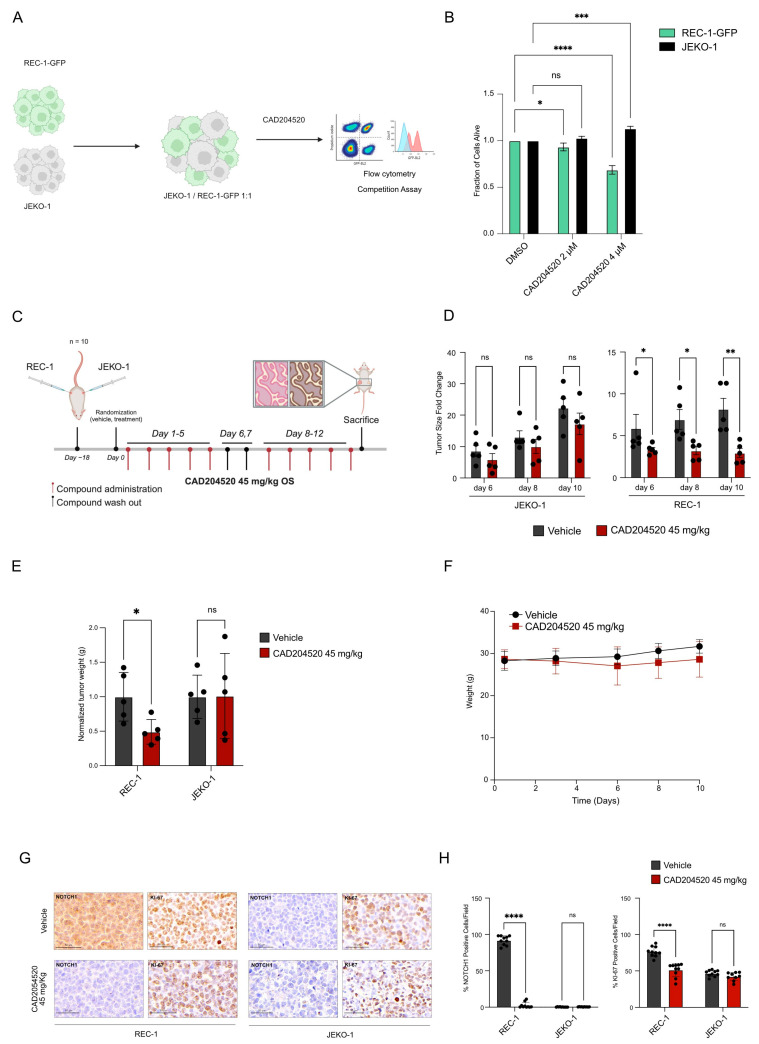
CAD204520 preferentially inhibits cells with *NOTCH1* PEST mutations: (**A**) Outline of the cell-based competition assay: REC-1 cells were transduced with green fluorescent protein (GFP). REC-1-GFP+ cells were sorted and co-cultured in a 1:1 ratio with JEKO-1 cells, then treated with CAD204520 at various concentrations for 72 h. (**B**) Normalized effects of CAD204520 on cell viability in co-cultured REC-1-GFP+ and JEKO-1 cells treated for 72 h. Statistical significance was determined by a two-way ANOVA. Error bars represent ± SD of a minimum of three replicates (* *p* < 0.05; *** *p* < 0.001; **** *p* < 0.0001). (**C**) Design of the in vivo CAD204520 study: ten NSG mice were subcutaneously injected with REC-1 cells in the left flank and JEKO-1 cells in the right flank. On day 0, mice were randomized into two groups, with the former receiving CAD204520 at 45 mg/kg (day 1–5 “on”; day 6–7 “off”; day 8–12 “on”) via oral gavage, and the latter receiving the vehicle. (**D**) Effect of CAD204520 administration on JEKO-1 and REC-1 tumor size fold change at different time points (mean ± SD of the five different mice treated with the vehicle or CAD204520). Statistical significance was determined using a non-parametric *t*-test (* *p* < 0.05; ** *p* < 0.01). (**E**) Effect of CAD204520 administration on JEKO-1 and REC-1 tumor weight at sacrifice (mean ± SD of the five different mice treated with the vehicle or CAD204520). Statistical significance was determined using a non-parametric *t*-test (* *p* < 0.05). (**F**) Effect of daily administration of 45 mg/kg of CAD204520 or the vehicle on body weight. (**G**) Immunohistochemical analysis of REC-1 and JEKO-1 tumor masses in the murine model treated with the vehicle or CAD204520 at 45 mg/kg for 10 administrations. The tumor masses from all mice were examined. Formalin-fixed, paraffin-embedded tissue sections were stained with NOTCH1 and Ki-67 antibodies. Scale bars: 50 µm. Representative results for one control animal and one CAD204520-treated animal are shown. (**H**) Quantification of NOTCH1 and Ki-67 protein expression of the immunohistochemical analysis shown in Figure 3G (**** *p* < 0.0001).

To further validate the activity of CAD204520 in *NOTCH1* PEST-mutated tumors, we established a flow cytometry competition assay using two MCL cell lines (REC-1 and JEKO-1) characterized by opposite *NOTCH1* mutational status. The choice of this model is related to the absence of commercially available CLL cell lines carrying *NOTCH1* PEST mutation. We transduced the REC-1 cell line carrying *NOTCH1* PEST mutation with a green fluorescent protein (GFP) and we treated REC-1-GFP and JEKO-1, cultured in a 1:1 ratio, at different CAD204520 concentrations (Figure 3A). As shown in Figure 3B, mutated cells displayed a more pronounced effect of CAD204520 compared to WT cells, thereby replicating the findings described earlier in the clinical sample setting.

In the past, we demonstrated that short-term exposure to CAD204520 led to a reduction in leukemia burden in a preclinical T-ALL model in vivo [14]. Here, we aimed to expand upon this discovery by investigating its applicability in a B-cell lymphoma model. Additionally, we sought to confirm the safety profile of CAD204520 with prolonged administration. To achieve this, we have established a subcutaneous xenograft model for comparative analysis. JEKO-1 and REC-1 cells were injected in the left and right flank of the same mouse, respectively. A total of five mice per group received ten doses of the vehicle or CAD204520 at 45 mg/kg by oral gavage. The administration was daily for 12 days including a 2-day washout period after the initial 5 days of treatment (Figure 3C).

We observed a significant reduction in REC-1 tumor size and weight starting six days after the start of treatment and at the end of the treatment, respectively, as illustrated in Figure 3D,E. Notably, the treatment exhibited excellent tolerability, with no major toxicities, including weight loss, as shown in Figure 3F. In PEST-mutated tumors, the immunohistochemical analysis of tumor samples obtained from treated mice indicated a consistent reduction in N1-ICD expression and proliferation, as evidenced by Ki-67 staining. In contrast, there was no significant change in NOTCH1 expression or proliferation rate in JEKO-1 tumors, as depicted in Figure 3G,H.

These promising preclinical in vivo model results, which align with our in vitro observations, strengthen our hypothesis that CAD204520 may be a potential candidate for improving the effectiveness of current drug treatments in patients with *NOTCH1* PEST mutations.

### 2.4. CAD204520 Increases the Effect of Venetoclax–Ibrutinib Combination Treatment in NOTCH1 PEST-Mutated Samples

Ibrutinib, an inhibitor of the Bruton tyrosine kinase (BTK), and venetoclax, an inhibitor of B-cell lymphoma-2 protein (BCL-2), were recently approved in combination by the European Medicines Agency (EMA) and the Food and Drug Administration (FDA) for the treatment of adult patients with CLL, offering a potential standard treatment for CLL and hopefully for MCL patients in the future [24,25]. However, it is important to note that CLL remains an incurable disease, and the presence of *NOTCH1* mutations is associated with an unfavorable outcome in both CLL and MCL patients.

For this reason, we have explored an approach involving the use of CAD204520 to enhance the response of the venetoclax–ibrutinib combination in patients carrying *NOTCH1* PEST mutations.

**Figure 4:**
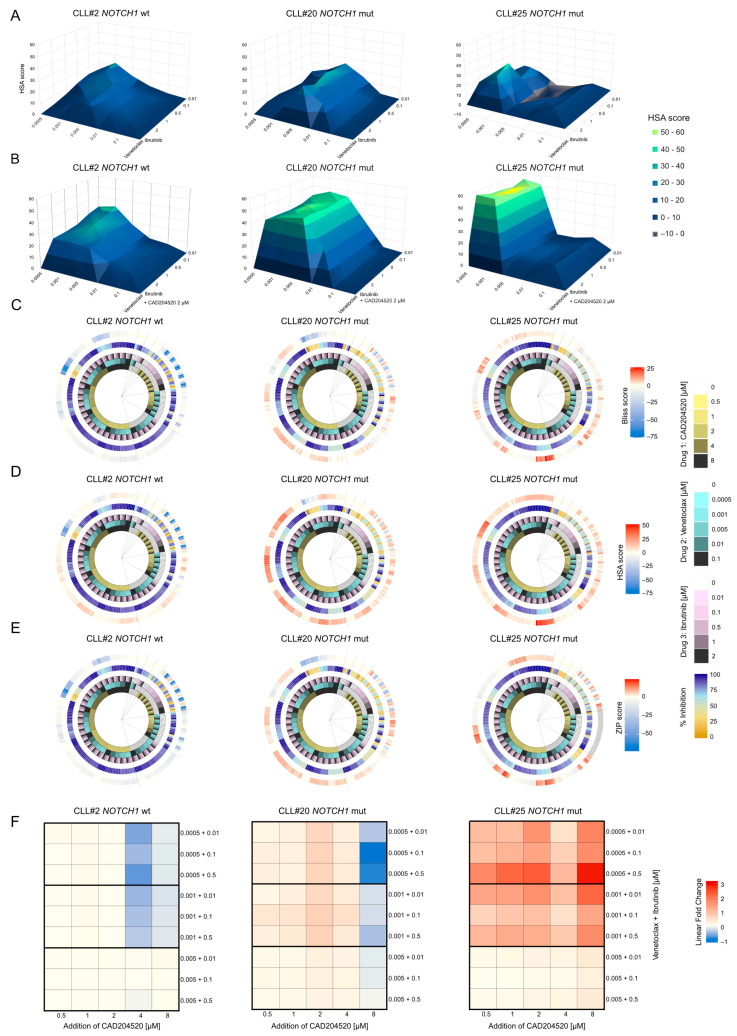
CAD204520 increases the effect of venetoclax–ibrutinib treatment in *NOTCH1* PEST-mutated samples: (**A**) Volcano surface plots of primary CLL samples with *NOTCH1* WT (CLL#2) and *NOTCH1* PEST mutation (CLL#20 and CLL#25) treated with venetoclax and ibrutinib. Each point represents an independent measurement. The plots illustrate the HSA analysis generated using the Combenefit script in MATLAB R201. The colorimetric scale represents the level of drug antagonism or synergism. (**B**) Volcano surface plots of primary CLL samples with *NOTCH1* WT (CLL#2) *NOTCH1* PEST mutation (CLL#20 and CLL#25) treated with venetoclax and ibrutinib plus 2 µM of CAD204520. Each point represents an independent measurement. The plots illustrate the HSA analysis generated using the Combenefit script in MATLAB R201. The colorimetric scale represents the level of drug antagonism or synergism. (**C**) Circular plots of 3-drug combinations in a *NOTCH1* WT primary sample (CLL#2) and two *NOTCH1*-mutated primary samples (CLL#20 and CLL#25). The innermost rings represent the 5 drug concentrations in 3 color gradients (CAD204520 = yellow, venetoclax = cyan, ibrutinib = pink). The fourth ring represents the effect in terms of inhibition percentage for any given combination. The outermost ring represents a harmonized synergy score performed with the Bliss model. Red indicates a positive synergistic score (pointing toward synergy), while blue represents a negative score (pointing toward antagonism). Zero indicates the absence of interaction. (**D**) Circular plots of 3-drug combinations following the same format as described in (**C**). The outermost ring represents a harmonized synergy score performed with the HSA model. (**E**) Circular plots of 3-drug combinations following the same format as described in (**C**). The outermost ring represents a harmonized synergy score performed with the ZIP model. (**F**) Heatmaps of samples CLL#2, CLL#20, and CLL#25 (from left to right) displaying the linear fold change between combinations of venetoclax-ibrutinib-CAD204520 (columns) compared to venetoclax-ibrutinib combinations (rows). Each cell contains the fold change between the 3-drug combination effect and the 2-drug combination effect at the same doses of the first 2 drugs. A positive fold change indicates a gain in inhibition of the 3-drug combination compared to the 2-drug combination, while a negative fold change indicates a loss of inhibition of the 3 drugs compared to the combination without CAD204520.

First, we treated both WT and *NOTCH1*-mutated samples with increasing concentrations of ibrutinib and venetoclax. Subsequently, we added a constant concentration of CAD204520 (2 µM) to the previously treated cells. The combination with CAD204520 resulted in a synergistic effect, observed in the *NOTCH1*-mutated samples, consistent with our prior findings (Figure 4A,B).

Next, we expanded the range of concentrations tested to assess the synergistic/antagonistic status of venetoclax-ibrutinib-CAD204520 combinations in a three-drug synergistic assay. This produced more than 200 combinatorial points interpolated from five concentrations of each drug. To assess the contribution of CAD204520 to venetoclax–ibrutinib combinations, we computed three different harmonized synergy scores (highest single agent: HSA; Bliss; and zero interaction potency: ZIP) for three different primary samples (one WT and two *NOTCH1*-mutated samples). *NOTCH1* PEST-mutated samples showed a stronger synergistic signal when compared to WT in all synergy scores. Furthermore, the non-mutated sample showed more combinations resulting in an antagonistic effect pointing to a lower inhibitory strength of venetoclax-ibrutinib-CAD204520 (Figure 4C–E).

Although synergy scores can indicate whether a drug combination induces synergistic or antagonistic effect, they do not quantify the gain or loss of inhibition when comparing the three-drug combination (venetoclax-ibrutinib-CAD204520) to the two-drug combination (venetoclax-ibrutinib). Therefore, to further dissect the difference between *NOTCH1* PEST-mutated and WT samples, we computed a linear fold change between the inhibition percentage of venetoclax-ibrutinib-CAD204520 combinations and those of venetoclax-ibrutinib at the same concentrations. Both mutated samples showed a linear fold change ≥ 1 (gain of inhibition) when venetoclax was ≤ 0.001 µM regardless of the ibrutinib dose (0.01 µM, 0.1 µM, and 0.5 µM), especially when CAD204520 was in the 0.5–4 µM range. On the opposite side, the WT sample showed a fold change close to zero, indicating the lack of difference in inhibition between combinations with and without CAD204520. In addition, the WT sample showed a negative fold change (CAD204520 4 µM and venetoclax ≤ 0.001 µM), indicating a loss of inhibition consequently CAD204520 addition to venetoclax-ibrutinib combination (Figure 4F).

Our results support the idea that CAD204520 enhanced the effect of venetoclax-ibrutinib combinations synergy treatment in *NOTCH1* PEST-mutated samples while WT samples showed a very limited improvement.

## 3. Discussion

Recent developments in cancer target therapy have shown promising results by targeting the genetic mutations or proteins responsible for tumor growth, leading to more effective and less toxic treatments. For example, patients with acute myeloid leukemia (AML) carrying the *FLT3* mutation may receive a targeted therapy like midostaurin or gilteritinib to inhibit the activity of the mutated FLT3 protein, potentially leading to improved outcomes and reduced side effects compared to traditional chemotherapy [26,27]. This paradigm appears to hold promise for all the mutations occurring in enzyme regulation in hematopoietic differentiation [28,29] and metabolism [30], but it is certainly less applicable to mutations involving transcription factors [31].

An example is the Notch signaling. A priori, NOTCH1 is not an ideal candidate for canonical drug targeting due to its involvement in various biological processes and cell types. This wide-ranging influence poses a potential limitation to developing effective anti-Notch1 therapies, as targeting NOTCH1 in non-leukemic cells could lead to adverse and toxic effects [4]. However, this is not the case. *NOTCH1* has been established as an oncogenic driver in several tumor models such as T-ALL [2], CLL [32], MCL [6], and a wide range of solid tumors [4], where activation recurs in different phases of the disease, both at diagnosis and relapse [2,33,34]. Canonical Notch1 signaling requires, multiple proteolytic cleavages such as S1 by a furin-like convertase in the endoplasmic reticulum/*trans*-Golgi compartment [35,36], a second cleavage (S2) within the NOTCH juxta-membrane extracellular domain mediated by zinc-dependent disintegrin and metalloprotease (ADAM10 or 17) at the membrane surface [37,38] and finally a third cleavage (S3) mediated by the γ-secretase complex [39]. These cleavages have the potential for enzymatic inhibition. For example, well-known GSI compounds have been extensively investigated in clinical trials, yet with limited success thus far [40].

In addition to enzymatic targeting, three other approaches for Notch signaling inhibition are under exploration. The first involves blocking individual NOTCH receptors or ligands with targeted antibodies. For example, a humanized antibody targeting NOTCH1, OMP-52M51 (brontictuzumab), has demonstrated efficacy in the inhibition of DLL4-mediated cleaved-NOTCH1 overexpression in pre-clinical studies [41,42] and has entered phase I trials for solid tumors and relapsed/refractory (R/R) lymphoid malignancies (NCT01778439, NCT01703572). The second approach relies on the binding inhibition of N1-ICD and its transcriptional complex [43]. The most advanced example in this case is CB-103, a small molecule investigated in Notch-driven cancers [44,45,46]. CB-103 showed good efficacy in *NOTCH*-mutated solid tumors and an acceptable safety profile in a phase I clinical trial [47]. The third approach depends on NOTCH1’s requirement to undergo cellular trafficking before relocating to the nucleus and initiating a transcriptional signal. In this context, small molecules targeting SERCA [12] or other ion channels [48,49] serve as prototypes for this therapeutic avenue. CAD204520, for example, showed an excellent safety profile, and a promising therapeutic index in preclinical models of T-ALL [14]. Previously, we collaboratively demonstrated that tumors with PEST mutations respond to ion channel modulators, such as the Ca^2+^/Na^2+^ pump inhibitor bepridil [49]. Thapsigargin, bepridil, ionomycin, salinomycin, and others were all initially identified through a gene-expression-based screen [11,50] designed to discover modulators of mutated Notch transcriptional programs in T-ALL. The repurposing effort of using these small molecules in tumors with *NOTCH1* mutations other than T-ALL, suggests that targeting Notch1 trafficking [11,51] could be equally effective across various tumor types [13]. In this sense, the activity of CAD204520 in CLL and MCL carrying PEST mutations does not surprise. However, what could not have been predicted is the fact that these mutations enhance the sensitivity to SERCA inhibitors compared to WT cases both in vitro and in vivo.

Targeting PEST domain mutation in hematological malignancies has been investigated in a few studies. The GSI PF-03084014 induces apoptosis in leukemic CLL cells carrying *NOTCH1* mutations, an effect potentiated by fludarabine [52]. Similarly, CLL xenotransplant models treated with bepridil significantly reduced tumor infiltration with no remarkable toxicity nor activity on NOTCH2 WT protein [49]. In MCL, the only clinically tested compound is the monoclonal antibody brontictuzumab. However, the preclinical activity of brontictuzumab in MCL cell lines was modest both in vitro and in vivo and similar to a minor clinical effect in MCL patients treated in the phase I study [42,53]. No clinical trials evaluating GSI in MCL are currently ongoing.

Furthermore, none of the previously mentioned studies performed a direct comparison of the effects of Notch1 inhibitors in mutated and non-mutated models in a head-to-head study. Such a comparison is crucial, considering the involvement of WT Notch signaling in some tumor types [54]. Achieving WT inhibition will necessitate dose adjustments, different schedules, or combinatorial approaches to effectively target WT polypeptides.

Besides Notch1, several other signaling pathways and small molecules have dominated the last ten years in research on cancer-carrying PEST domain mutations. This is the case of B-cell receptor (BCR)-associated kinases, such as BTK, phosphoinositide 3-kinases (PI3K), and the anti-apoptotic protein BCL-2 [55,56,57,58]. Randomized clinical trials demonstrated impressive activity of ibrutinib and novel BTK inhibitors for the treatment of R/R disease [59,60,61], del(17p) CLL patients [62], and de novo or R/R MCL patients [63,64]. In parallel, venetoclax was the first BCL-2 inhibitor to enter routine clinical practice. In a phase I study, venetoclax induced durable responses in 79% of patients with R/R CLL, including complete remissions in 20% of patients [65]. Given their impressive effect, it is a reasonable strategy to combine the two molecules. In the setting of combinatory therapy venetoclax plus ibrutinib, a phase II non-randomized trial (NCT02756897) in treatment-naive patients [66] has shown a 3-year progression-free survival (PFS) of 93%, including durable activity in del(17p)/*TP53*-mutated CLL. Interestingly, the combinatory treatment with ibrutinib plus venetoclax has shown encouraging clinical activity in early phase studies, reaching a phase III SYMPATICO trial with strong efficacy in patients with R/R MCL [25].

In the scenario where the majority of CLL patients respond to ibrutinib or venetoclax, *NOTCH1*-mutated patients still represent an aggressive subgroup of the disease, as *NOTCH1* mutation showed to be an independent predictor of survival and Richter transformation [16,67,68]. Based on this assumption, one question arises: when and how to incorporate Notch inhibitors? One possible answer is to consider cases that have relapsed or are refractory to therapy, or cases that are progressing despite ongoing therapy [69]. Combination therapy appears to be a potential strategy, as there is evidence, for example, that GSI enhances the anti-leukemic activity of ibrutinib in CLL cells by down-regulating the Notch1 and c-Myc pathways [70]. In addition, ibrutinib treatment was shown to downregulate NOTCH over time as part of the downstream pathway of the BCR [71]. Finally, although the presence of a mutation does not appear to negatively impact the efficacy of ibrutinib in terms of disease progression outcomes [72], other findings correlate *NOTCH1* mutation with reduced redistribution of lymphocytosis and nodal shrinkage, responsible for partial responses and early relapses [73]. For MCL instead, the answer is simpler given the urgent need for new approaches for R/R cases or cases not eligible for CAR-T therapy [74].

However, mimicking this complex setting requires building a feasible toolbox for the analysis of drug synergy with more than two compounds. This effort presents several challenges, for example, the lack of tools capable of handling combinations involving N-drugs (N > 2), which limits the information retrievable from these combination experiments. Another limiting factor involves the strategy employed by current methods, where synergy scores for combinations of three drugs are computed by comparing the effect of the triplet with the effects of each single drug, without considering any comparison between the two-drug combinations and the triplet [75,76]. A method that would eliminate these issues involves computing synergy scores for pairwise combinations and using them to gain information about higher-order combinations. However, this type of approach has been reported to rarely show synergy, whereas antagonism is more common [77,78,79,80]. This arises from the inability of pairwise comparisons to predict higher-order interactions [76].

To overcome existing analytical limitations, we decided to use established but methodologically limited, synergy scores (HSA, Bliss, and ZIP). Additionally, to obtain a direct measure of gain or loss of inhibition, we integrated the results with the implementation of a simple quantitative method based on the linear fold change between venetoclax-ibrutinib-CAD204520 and venetoclax-ibrutinib combinations. Collectively, our data suggest that a low concentration of venetoclax may be sufficient to prime the cells to death when co-treated with the BTK inhibitor ibrutinib and the SERCA inhibitor CAD204520. This approach may reduce the requirement for higher concentrations of venetoclax, which can potentially give rise to BCL-2-resistant clones. The addition of CAD204520, in turn, can have a sustained positive impact on controlling the proliferation of leukemic cells.

In conclusion, our work positions SERCA inhibitors as potential modulators of the Notch signaling characterized by PEST mutations such as CLL and MCL and supports the development of novel strategies with complex matrices of drug-drug combinations in preclinical cancer-related studies.

## 4. Materials and Methods

### 4.1. Cell Lines

The human cell lines ALL-SIL, SKW-3/KE-37, CTV-1, MEC-1, JEKO-1, REC-1, and Granta-519 were purchased from the Leibniz Institut DSMZ-German collection of microorganisms and cell cultures (Germany). Cells were cultured in RPMI 1640 (#MT10040CV, Thermo Fisher Scientific, Waltham MA, USA) with 10% or 20% fetal bovine serum (FBS) (#10270–106, Thermo Fisher Scientific), 1% penicillin-streptomycin (P/S) (#3MT30002CI, Thermo Fisher Scientific), and 1% of MEM Non-Essential Amino Acids Solution (100X) (#11140050, Thermo Fisher Scientific), 2 mM L-Glutamine (#25030-081, Thermo Fisher Scientific), and 1% HEPES Buffer 1M (#MS013D1006, Biowest, Nuaillé, France). Granta-519 was maintained in DMEM (#11960-044, Thermo Fisher Scientific) with 20% FBS, 1% P/S, and 2 mM L-Glutamine (#25030-081, Thermo Fisher Scientific). Cell lines were grown in a humidified incubator at 37 °C, and 5% CO_2_ and monitored for mycoplasma contamination.

### 4.2. Primary Samples

Chronic lymphocytic leukemia (CLL) cells derived from peripheral blood (PB) were obtained from patients with CLL under an approved protocol from the Department of Medicine and Surgery at Parma University Hospital (n.29785/13 July 2021), according to the declaration of Helsinki guidelines for the protection of human rights. Lymphocytes from PB samples were isolated through a density gradient centrifugation using Lympholyte Cell Separation Media (#CL-5020, EuroClone SpA, Pero, Italy) and cultured in IMDM (#12440-053, Thermo Fisher Scientific) with 20% FBS, and 1% P/S.

### 4.3. Karyotype Analysis and Fluorescence In Situ Hybridization

Primary peripherical blood samples were cultured for 72 h in RPMI 1640 with 20% FBS, 1% P/S, and ChromoLympho-B Proliferation MIX (with CpG-oligonucleotide DSP30 plus IL-2) (#EKAMP010M, Euroclone SpA), to increase the leukemic B lymphocyte proliferation and improve the mitotic rate. Cell media was supplemented with 0.1 μg/mL of colcemid (#15212012, Thermo Fisher Scientific,) for 2 h, followed by incubation in a hypotonic solution (0.075 M KCl). Cells were fixed in a 3:1 methanol (#322415, Sigma-Aldrich, St. Louis, MO, USA) and acetic acid glacial fixative solution (#A6283, Sigma-Aldrich) and spread on top of Superfrost Plus microscope slides (#10149870, Thermo Fisher Scientific). For the karyotype analysis, chromosome banding was performed by quinacrine (Q-banding) staining. A minimum of 20 metaphases per sample were acquired using a Nikon Eclipse 80i microscope (Nikon Instruments, Inc., Melville, NY, USA) and analyzed using NIS element software V3.8 (Nikon Instruments, Inc.). For the fluorescence in situ hybridization (FISH) analysis, 10 μL of XL ATM/TP53 (#D-5046-100-OG, Metasystems, Altlussheim, Germany) or set probe” XL DLEU/LAMP/12cen (#D-5055-100-TC, Metasystems) were incubated at 37 °C for 12–16 h after a phase of DNA dehydration with ethanol-scale incubation (75%–85%–100%)and DNA denaturation (75 °C, 5 min). Slides were washed once with 0.4× saline sodium-citrate/0.3% NP40 buffer at 73 °C, followed by 4× SSC/0.1% NP-40 at ambient temperature. DNA was counterstained with 4′,6-diamidino-2-phenylindole (DAPI; #10236276001, Sigma-Aldrich) before microscope analysis (Eclipse 80i microscope, Nikon Instruments, Inc.). Two hundred interphase nuclei were analyzed for each patient and DNA abnormalities were defined starting from a 5% cutoff for each probe. For the detection of del(17p), a cutoff of 20% interphase nuclei was adopted, in line with previous findings [81].

### 4.4. Next-Generation Sequencing

DNA was extracted using a Maxwell^®^ 16 DNA purification kit (#AS1010, Promega Corporation, Madison, WI, USA), following the manufacturer’s instructions. The concentration and purity of the DNA samples were determined with a Qubit 4 fluorometer (#33226, Thermo Fisher Scientific). Primary samples were sequenced using the Sophia Lymphoma Solution^TM^ kit (#CS.2205.0103-00, Sophia Genetics SA, Rolle, Switzerland). Library preparation and sequencing were performed on a MySeq system (Illumina Inc., San Diego, CA, USA) following the manufacturer’s instructions. Data were analyzed with Sophia DDM^®^ software version 5.10.11.1 (Sophia Genetics SA). A cutoff VAF of ≥10% for *TP53* gene mutations was adopted, in line with the European Research Initiative on CLL (ERIC) recommendations [20].

### 4.5. Western Immunoblot and Antibodies

Whole-cell protein lysates were extracted using 1× Cell Lysis buffer (#9803S, Cell Signaling Technology, Danvers, MA, USA) with Protease/phosphatase Inhibitor Cocktail 100X (#58725, Cell Signaling Technology, Danvers, MA, USA). Cells were lysed on ice for 30 min with gentle stirring and centrifuged at 14,000 RPM for 10 min at 4 °C. Protein lysates were quantified using Bio-Rad Protein Assay Dye Reagent (#5000006, Bio-Rad Laboratories, Hercules, CA, USA) and the total lysate/sample was loaded for SDS-PAGE analysis. Primary antibodies for immunoblot detection were purchased from Cell Signaling Technology: NOTCH1 XP (#3608S) and cleaved NOTCH1 (#4147S). Loading controls were performed with antibodies specific for β-Actin (#3700S and #4970S). IRDye 680LT Goat anti-Mouse IgG (#925-68020, LI-COR Biosciences, Lincoln, NE, USA) and IRDye 800CW Goat anti-Rabbit IgG (#925-32211, LI-COR Biosciences) were used as secondary species-specific antibodies. Membranes were detected using the LI-COR Odyssey imaging system (LI-COR Biotechnology: Lincoln, NE, USA) and the Chemidoc MP Imaging System (Bio-Rad Laboratories).

### 4.6. Cell Treatment and Viability Assays

CAD204520 was obtained as a kind gift from WDB R&D Consulting (Copenhagen, Denmark). Venetoclax (S8048) and ibrutinib (S2680) were purchased from Selleck Chemicals (SelleckChem, Houston, TX, USA) and dissolved in DMSO, according to the manufacturer’s instructions. A total of 40,000 cells were arrayed in 96-well plates (Greiner Bio-One, St. Gallen, Switzerland) in a volume of 100 μL per well using the MultiDrop Combi Reagent Dispenser (Thermo Scientific). Cell treatment was performed with Tecan D300e (Tecan Group, Zurich, Switzerland). ATP-based cell viability was determined using the CellTiter-Glo viability assay (#G7573, Promega Corporation, Fitchburg, WI, USA) after 72 h of treatment. Luminescence was measured using a Victor X4 (Perkin Elmer, Waltham, MA, USA). Values for IC50 and the area under the curve (AUC) were calculated using GraphPad Prism 9 software (La Jolla, CA, USA). Death rate of cells after CAD204520 treatment was assessed using a flow cytometric assay, using the Attune NxT flow cytometer (Thermo Fisher Scientific). Cells were stained with Propidium iodide (PI) (#40017, Biotium, Inc. Landing Parkway Fremont, CA, USA) and human CD5 (#345781, Becton Dickinson Biosciences, Franklin Lakes, NJ, USA). The percentage of death cells was quantified acquiring a minimum of 10000 events. Data were processed with FlowJo V10 (Tree Star, LLC, Ashland, OR, USA) analytical software.

### 4.7. Drug Combination Treatment and Synergy Assessment

Cell solution (50 μL/well of 0.02 × 10^6^/mL) was dispensed in 384-well plates (#3570, Corning Life Sciences Plastic, Bedford, MA, USA) using Multidrop^TM^ Combi (#5840300, Thermo Fisher Scientific). Venetoclax, ibrutinib, and CAD204520 were dissolved in DMSO and added with a nanometric Tecan D300e dispenser. We tested ibrutinib and venetoclax both individually and in combinations for a total of twenty-five combinatorial points in three CLL primary samples. Each drug was tested in 5 concentrations, with or without 2 µM of CAD204520. Cell viability was assessed after 72 h of drug treatment using a CellTiter-Glo ATP assay. Analysis was performed with Combenefit MATLAB R201 [82], using the HSA synergy analysis. A color scale bar represents the level of drug antagonism or synergism. For the 3-drug combination, we assessed the synergy, using three primary CLL samples. Each drug was tested at five different concentrations. Cell viability was assessed after 72 h of drug treatment using a CellTiter-Glo ATP assay. Subsequently, data were imported into R (version 4.3.1). Before calculating synergy scores, we addressed situations where some viability values exceeded 1 (or 100%), as such instances can pose issues when computing synergy scores.

Therefore, viability data were re-scaled to set the maximum value to 1 without altering the minimum value in each sample using a generalized scaling formula. Moreover, this adjusted viability was converted into inhibition which was then used to compute three different harmonized synergy scores: HSA, Bliss, and ZIP implemented in the SynergyFinder Plus R package [83,84]. Each harmonized score is centered on zero with positive values pointing toward synergistic effect and negative values pointing to antagonistic effect; therefore, all scores can be compared to each other. Then, we computed a linear fold change between combinations of 3 drugs (venetoclax-ibrutinib-CAD204520) and 2 drugs (venetoclax-ibrutinib) in order to obtain a quantitative measure of the gain/loss induced by the addition of varying doses of CAD204520 to the venetoclax-ibrutinib combinations. A positive fold change value represents combinations in which the addition of CAD204520 caused an inhibition improvement while a negative fold change value represents combinations in which the addition of CAD204520 resulted in inhibition dampening. All plots were made using R and the ggplot2 package [85].

### 4.8. Cell Competition Assay

REC-1 were transduced with a green fluorescent protein (GFP) lentiviral expressing vector and co-cultured with JEKO-1 cells in a 1:1 ratio in RPMI 1640, 10% FBS, 1% P/S, and 1% MEM Non-Essential Amino Acids. Three million cells per condition were treated with vehicle or CAD204520 at concentrations of 2 and 4 µM. Cells were incubated at 37 °C for 72 h, washed in PBS, and then stained with PI and human CD5 for 15 min. Fluorescent signal was detected by flow cytometry and a minimum of 10,000 events were collected for each biological condition. Data were processed by FlowJo V10 analytical software.

### 4.9. In Vivo Study

Ten non-irradiated 6 to 7-week-old non-obese diabetic (NOD)-scid IL2rγ(null) (NSG) mice (Charles River Laboratories, Wilmington, MA, USA) were used for the in vivo study. Ten million REC-1 cells and JEKO-1 cells, dissolved in 250 μL saline solution, were subcutaneously injected into the left and right flank of the same mouse, respectively. Once the tumor was established and palpable on both sides, mice were divided into vehicle and CAD204520 treatment groups, respectively. NSG mice received 45 mg/kg CAD204520 (Tween80 0.5% *w*/*v*; hydroxypropyl methylcellulose (HPMC) 1% *w*/*v*) or vehicle by oral gavage from day 1 to day 5 and from day 8 to day 12. Weight was monitored every 2 days. The anti-tumor activity of CAD204520 was assessed by measuring the REC-1 and JEKO-1 tumor volume by caliper measurement at days 0, 3, 6, 8, and 10, and by quantification of NOTCH1 (#PA5-99448; CleavedVal1744; Thermo Fisher Scientific Invitrogen) and KI-67 (#R626; Agilent, Santa Clara, CA, USA)-positive cells in formalin-fixed, paraffin-embedded tumor sections. Images were acquired at different magnifications using a Leica DM750 microscope (Leica Microsystems, Wetzlar, Germany). The studies were carried out under an approved protocol n°682/2019-PR at the University of Parma.

## Data Availability

Data are contained within the article and Supplementary Materials.

## Supplementary Materials

The supporting information can be downloaded at: https://www.mdpi.com/article/10.3390/ijms25020766/s1.
